# D-Limonene Affects the Feeding Behavior and the Acquisition and Transmission of Tomato Yellow Leaf Curl Virus by *Bemisia tabaci*

**DOI:** 10.3390/v16020300

**Published:** 2024-02-15

**Authors:** Yan Wei, Liming Gao, Zhanhong Zhang, Kailong Li, Zhuo Zhang, Deyong Zhang, Jianbin Chen, Jing Peng, Yang Gao, Jiao Du, Shuo Yan, Xiaobin Shi, Yong Liu

**Affiliations:** 1 Institute of Plant Protection, Hunan Academy of Agricultural Sciences, Changsha 410125, China; weiyan960619@163.com (Y.W.); liminggao124@163.com (L.G.); lannuolkl@foxmail.com (K.L.); lionkingno.1@163.com (Z.Z.); dyzhang78@163.com (D.Z.); chenjianbin89@126.com (J.C.); pengjing_617@126.com (J.P.); lansetianji519@126.com (Y.G.); dujiao1234xy@163.com (J.D.); yanshuo189@hotmail.com (S.Y.); 2 Yuelushan Laboratory, Changsha 410215, China; 3 Institute of Vegetable Crops, Hunan Academy of Agricultural Sciences, Changsha 410125, China; hvizhanhong@163.com

**Keywords:** *Bemisia tabaci*, EPG, odorant-binding protein, fluorescence competitive binding assays, molecular docking

## Abstract

*Bemisia tabaci* (Gennadius) is an important invasive pest transmitting plant viruses that are maintained through a plant–insect–plant cycle. Tomato yellow leaf curl virus (TYLCV) can be transmitted in a persistent manner by *B. tabaci*, which causes great losses to global agricultural production. From an environmentally friendly, sustainable, and efficient point of view, in this study, we explored the function of d-limonene in reducing the acquisition and transmission of TYLCV by *B. tabaci* as a repellent volatile. D-limonene increased the duration of non-feeding waves and reduced the duration of phloem feeding in non-viruliferous and viruliferous whiteflies by the Electrical Penetration Graph technique (EPG). Additionally, after treatment with d-limonene, the acquisition and transmission rate of TYLCV was reduced. Furthermore, BtabOBP3 was determined as the molecular target for recognizing d-limonene by real-time quantitative PCR (RT-qPCR), fluorescence competitive binding assays, and molecular docking. These results confirmed that d-limonene is an important functional volatile which showed a potential contribution against viral infections with potential implications for developing effective TYLCV control strategies.

## 1. Introduction

*Bemisia tabaci* (Gennadius) (Hemiptera: Aleyrodidae) is one of the most important invasive pests, causing huge economic losses worldwide [1,2]. *B. tabaci* has a wide range of hosts with more than 500 plant species, such as peppers, tomatoes, tobacco, grains, and beans [3]. The two cryptic species of *B. tabaci*, MEAM1 (B) and MED (Q), are highly invasive and have replaced other native cryptic species in many areas of the world, causing serious harm [4,5]. *B. tabaci* damages crops by removing phloem sap, secreting honeydew on leaves, and transmitting plant viruses [3,6]. For example, whiteflies can circularly transmit the tomato yellow leaf curl virus (TYLCV) in Begomoviruses [7]. TYLCV is a single-stranded circular DNA virus. It can infect a variety of plants, such as peppers (*Capsicum* species), gourds (*Cucumis* species), beans (*Phaseolus vulgaris*), eustoma (*Eustoma grandiflora*), and tomatoes (*Lycopersicon esculentum* Mill.) [8,9,10,11,12]. Typical symptoms of infected tomato plants are leaf curling, wilting, intervein yellowing, stunting, and degeneration, resulting in reduced production and serious economic losses [13,14,15,16]. TYLCV in tomatoes is difficult and expensive to manage, whether in open-field production or structural cultivation [17]. There are many methods to control TYLCV, such as controlling the population of whiteflies, killing intermediate hosts (weeds), and changing the planting season. However, a single method tends to increase the control difficulty and is not frequently effective [8]. Therefore, reducing the population and migration of the vector whiteflies and preventing their transmission have become important difficulties in control. Although various methods are used, it is still difficult to control this key pest, and its management relies primarily on using insecticides [6]. However, the indiscriminate use of insecticides causes resistance development. It potentially harms non-target insects and the environment [18]. From environmentally friendly, sustainable, and efficient points of view, more methods have been used to control *B. tabaci*, such as plant volatiles, natural insecticides, and biological control [19,20,21].

Several studies have indicated that volatiles are the key factor influencing host preference in *B. tabaci* [22,23,24]. By identifying the functional volatiles, the preference of *B. tabaci* for host plants can be changed to further control their numbers on host plants. Therefore, an increasing number of functions of volatiles have been revealed, choosing suitable volatiles and then using “pushing” (repellent) and “pulling” (attractant) to provide new strategies for *B. tabaci* population control [24]. Insects rely on a sensitive and complex olfactory system to detect chemicals to complete their physiological behaviors [25,26,27]. Also, odorant-binding proteins (OBPs) are important components of the olfactory system and the first step in the signal chain of odorant perception [28]. Determining the odorant-binding proteins that bind to plant volatiles could help increase the control effect on pests. For example, ApisOBP3 and ApisOBP7 played key roles in aphid response to the alarm pheromone (E)-β-farnesene, a target gene that attenuates signal perception in aphids [29]. (E)-β-farnesene could be detected by *Eupeodes corollae* via EcorOBP15, an important basis for *E. corollae* to prey on aphids [30]. Moreover, AlucOBP5 combined with cis-3-hexenal and phenylacetaldehyde to help *Apolygus lucorum* locate hosts and find nectar. These target genes provide new strategies for *A. lucorum* control [25]. Currently, there is no in-depth information on the target receptor of *B. tabaci*, especially virus-infected *B. tabaci*, for repellent volatiles, which could help manage it effectively. At first, we found that the number of *B. tabaci* on three plants, *Apium graveolens* (*A. graveolens*), *Agastache rugosa* (*A. rugosa*), and *Coriandrum sativum* (*C. sativum*), in the laboratory was very low, so we chose them as test plants to verify their repellent effect on *B. tabaci*.

In our study, *A. graveolens*, *A. rugosa*, and *C. sativum* were found to repel healthy and TYLCV-infected *B. tabaci*. Moreover, d-limonene was identified as the most crucial functional volatile compound among their volatile components. Based on these findings, we hypothesized that d-limonene performs more important biological functions besides its evasive effect on *B. tabaci*. The following experiments were conducted to validate this hypothesis: (1) The preference for d-limonene among non-viruliferous and viruliferous whiteflies was determined. (2) The effects of d-limonene on feeding behavior and TYLCV acquisition and transmission of whitefly were investigated. (3) The target gene of OBPs in nonviruliferous and viruliferous whiteflies that recognizes d-limonene was identified, and the BtabOBP3 was cloned and analyzed to determine their binding capabilities. (4) Molecular docking techniques were employed to predict the key binding sites of d-limonene with BtabOBP3.

## 2. Materials and Methods

### 2.1. Analysis of Key Volatile Compounds in A. graveolens, A. rugosa, and C. sativum and Their Impact on the Preference of B. tabaci

#### 2.1.1. *B. tabaci* MEDs and Plant Rearing

*B. tabaci* MED used in this experiment was presented by Dr. Youjun Zhang from Chinese Academy of Agricultural Sciences. Healthy adults were reared on tomato plants (*Solanum lycopersicum* Mill. cv. Zuanhongmeina) for more than 6 generations to establish a population with no pesticide exposure. *A. graveolens*, *A. rugosa*, and *C. sativum* seeds were purchased from the market. The tomato seeds were purchased from the Institute of Vegetable Crops, Hunan Academy of Agricultural Sciences. All seedlings were planted in a greenhouse. TYLCV-infected tomato was characterized by leaf curl and shrinking (Figure S1), and tomatoes were determined by reverse transcription (RT)-PCR using gene-specific primers (Table S1). Healthy adults had fed on TYLCV-infected tomato plants for 48 h to obtain viruliferous whiteflies [31,32]. The temperature of the rearing chamber was kept at 26 ± 2 °C. The relative humidity was controlled at 55 ± 5%, and the photoperiod was set to 14 L/10 D.

#### 2.1.2. The Preference of *B. tabaci* MEDs Assessed by Y-Tube Olfactometer Behavioral Experiments

The preference of *B. tabaci* MED adults (non-viruliferous and viruliferous) for three plants at a specific time was evaluated by a Y-tube olfactometer. One branch of the Y-tube was placed with the plant, and the other was treated with air as a control. The equipment was ventilated for 30 min to stabilize the air volume at 0.6 L/min. The lighting conditions of the Y-tube were the same during all tests. The central tube is 40 cm, the two branch arms are 30 cm, and the angle between the two arms is 60°. Each *B. tabaci* MED was put into the main arm after 2 h of starvation. The observation time for each *B. tabaci* MED was 3 min, and the choice of each plant in any branch was observed when the tested *B. tabaci* MEDs moved more than 1/3 into one of the two arms. Otherwise, it was regarded as no choice [33]. Each adult was selected only once. A total of 10 whiteflies were used, 10 times in each group, and six groups were repeated. The Y-tube was washed with 95% ethanol after the test. After each test, the positions of the treatment and control were changed to eliminate the influence of the two side arms of the Y-tube. The numbers of *B. tabaci* were counted in two side arms, and the repellent rate was calculated by the following formula [34]:

$$\begin{array}{l} Repellent\;rate:\\ =\frac{B.\;tabaci\;in\;control\;arm\;-\;B.\;tabaci\;in\;treatment\;arm}{B.\;tabaci\;in\;control\;arm\;+\;B.\;tabaci\;in\;treatment\;arm}\times 100 \end{array}$$

Regarding d-limonene, the preference of *B. tabaci* MED adults (non-viruliferous and viruliferous) was also found by Y-tube olfactometer experiments. One branch of the Y-tube was placed with d-limonene (the concentrations ranged from 10^−1^ to 10^−6^ g/mL, diluted with n-hexane). The configured 2 mL compound was transferred to the filter paper and volatilized in the test tube. N-hexane was selected as the control when evaluating *B. tabaci*’s selectivity to d-limonene. D-limonene and n-hexane were purchased from Macklin reagent Co., Ltd. (Shanghai, China). The remaining experimental methods were conducted following the procedures outlined in Section 2.1.2.

#### 2.1.3. Extraction and Identification of Volatile Compounds in *A. graveolens*, *A. rugosa*, and *C. sativum*

Gas chromatography–mass spectrometry (GC-MS) was used for the identification of volatiles. The plants grown in the greenhouse for 8 weeks were selected to extract and identify volatile compounds. Leaves from different parts (top, middle, and bottom) of each plant were collected, totaling 500 mg, and ground to a fine powder using liquid nitrogen. The samples were transferred to 2 mL centrifuge tubes and mixed with 1 mL of n-hexane. After vortexing for 30 s, they were transferred to brown sample vials using disposable sterile needles and a 0.22 μm bacterial filter and placed on ice as samples. Each experiment was conducted with three biological replicates [35].

For the GC, the starting injector temperature was 50 °C for 1 min, ramped at 5 °C/min to 240 °C for 2 min, and then ramped at 30 °C/min to 300 °C for 5 min. The MS electronic impact ionization energy was set to 71 eV. The ion source, MS quad, was set to 230 °C and 150 °C, and the mass scanning range was set to 50–650 *m*/*z* with 0.5 scans/s. Data were correlated with the mass spectra of these compounds, and the database was searched for similar compounds with same retention time and molecular mass. The column type used for GC-MS was HP-5MS quartz capillary column (30 m × 0.25 μm × 0.25 μm). All peaks were identified from their mass spectra by comparison with those present in NIST 11 libraries (McLafferty, F.W. Wiley Registry of Mass Spectral Data) [36].

### 2.2. Effects of D-Limonene on Stylet Activities, the Acquisition, and Transmission of TYLCV by B. tabaci

#### 2.2.1. Feeding Behavior of Nonviruliferous and Viruliferous Whitefly after Treatment of D-Limonene

The d-limonene was mixed with n-hexane to prepare a working solution of 10^−2^ g/mL, which was uniformly sprayed on the surface of tomato plants using a 10 mL sprayer. Tomatoes grown in the greenhouse for 4 weeks were used to measure feeding behavior. These tomatoes were used to record the feeding behavior of non-viruliferous and viruliferous whiteflies. The sexes were distinguished, and females were selected under a stereoscopic microscope (Figure S2). The newly emerged females from healthy tomatoes in 3–5 days (defined as non-toxic whiteflies) were fed on TYLCV-infected tomato plants, and viruliferous whiteflies were obtained 48 h later. Before the experiment, whiteflies were starved for 2 h [37].

Conductive silver glue was utilized to secure one whitefly onto a 12 μm gold wire electrode. The electrode was then connected to the EPG system, and the date of feeding behavior was acquired and analyzed using Stylet + for Windows software (EPG stylet + d, Wageningen, The Netherlands) [38]. All materials were placed inside a Faraday cage, and the test was carried out at the temperature of the rearing chamber from 10:00 to 18:00. EPG waveforms were categorized into four groups: np (non-probing behavior), C (showing the insect intercellular stylet pathway), E1 (phloem salivation), and E2 (phloem ingestion). Each group was set up with 10 biological replicates [39].

#### 2.2.2. Influence of D-Limonene on the Acquisition and Transmission of TYLCV by *B. tabaci*

As described in Section 2.2.1, female adults were fed on d-limonene-treated tomato plants (treatment group) or n-hexane-treated tomato plants (control group) for 12 h. Then, treated female adults were transferred to clip cages (50 whiteflies/cage) to starve for 2 h. Clip cages containing starved *B. tabaci* were placed on TYLCV-infected tomato plants (3 cages/plant) for virulence acquisition. After 6, 12, 24, 48, or 72 h of feeding, 100 whiteflies from each treatment were collected, and RNA was extracted from each whitefly. TYLCV was detected by RT-PCR and agarose gel electrophoresis, and TYLCV detection results were used to calculate the acquisition rate.

After 48 h of TYLCV acquisition, *B. tabaci* were placed in clip cages (1, 5, 10, 25, and 50 *B. tabaci*/cage) and fixed on healthy tomato plants (20 tomato plants/group). After 48 h of feeding, the clip cages were removed, and tomato plants were further cultivated without *B. tabaci*. After 14 days, TYLCV was separately detected in tomato plants by RT-PCR (Table S1) and agarose gel electrophoresis. Based on the TYLCV infectivity in different groups, the transmission rate was calculated. Four independent replications were performed for each experiment [32].

### 2.3. Binding Analysis of OBP Associated with D-Limonene Recognition in B. tabaci

#### 2.3.1. The Relative Gene Expression of BtabOBPs

The relative gene expression of *BtabOBP*s in non-viruliferous and viruliferous whiteflies after d-limonene treatment at 0, 6, and 12 h was determined by RT-qPCR. cDNA was quantified to 200 ng, combined with specific primers designed by Primmer 5 (Table S1). qPCR was performed on the qTOWER3G fluorescence quantitative PCR instrument. Fifty adult insects were used in each experiment, with three technical and three biological replicates. The relative expression of *BtabOBP*s was calculated using the comparative cycle threshold (2^−ΔΔct^) method [40].

#### 2.3.2. Competitive Fluorescence Binding Assay

The PCR product of *BtabOBP3* was cloned into a pEASY-T3 vector. After excising the vector, the target fragment was cloned into the expression vector pET-28b (+) for expression in the *BL21* (DE3) strain. *BtabOBP3* was induced with 1 mM isopropyl β-D-1-thiogalactopyranoside (IPTG) for 4 h at 37 °C. BtabOBP3 was expressed in the inclusion bodies. Inclusion bodies were denatured by 8 M urea. Recombinant proteins of BtabOBP3 were solubilized and refolded based on the reported methods [41]. Protein purification was performed with His-Tag purification resin column (LMAI Bio) and purified by 300 mM imidazole buffer. The purity and size of purified recombinant proteins were detected by SDS-PAGE. The protein concentrations were measured with bicinchoninic acid (BCA) Protein Assay Ki [42].

N-phenyl-1-naphthylamine (1-NPN) was used as the fluorescent probe. 1-NPN and d-limonene were diluted with methanol to 1 mM. BtabOBP3 (2 μM) of 2 mL was added to a 10 mm fluorescence cuvette. The scanning results showed that the optimal emission wavelength (Figure S5A) and excitation wavelength (Figure S5B) of BtabOBP3 are 337 nm and 278 nm. At the excitation wavelength of 277 nm, the fluorescence spectrum of BtabOBP3 binding to 1-NPN was recorded at 300–500 nm. 1-NPN (1 mM) was added to BtabOBP3 at concentrations ranging from 2 to 16 μM. The binding constant of 1-NPN to BtabOBP3 (K_1−NPN_) was calculated using GraphPad Prism Windows 5.00 software. 1−NPN was mixed equally with 2 μM BtabOBP3, then 1 mM d-limonene was successfully translated into the mixture. The excitation wavelength was set to 220 nm. The emission spectrum was recorded at 250–550 nm. The dissociation constant of d-limonene was calculated with the equation K_i_ = [IC_50_]/(1 + [1−NPN])/K_1−NPN_], where [1−NPN] is the free concentration of 1-NPN, and K_1−NPN_ is the dissociation constant of the protein/1−NPN [43].

### 2.4. Homology Modeling of BtabOBP3 and Molecular Docking with D-Limonene

The amino acid sequence of the protein was obtained from the NCBI. The 3D model of BtabOBP3 was constructed by AplhaFold, and the molecular docking was performed using AutoDock 4.2 software [44]. Then, the small structure molecules were downloaded from (https://pubchem.ncbi.nlm.nih.gov (accessed on 15 October 2022), and Chem3D was used for structure optimization. The entire protein was wrapped in a docking box, and 200 conformations were searched. The lowest-energy conformation was selected and visualized with pymol.

### 2.5. Data Analysis

All the statistical data analyses were performed by SPSS 22.0 (SPSS Inc., Chicago, IL, USA). The independent samples *t*-test (* *p* < 0.05, ** *p* < 0.01, *** *p* < 0.001, ^ns^ *p* > 0.05) and ANOVAs followed by Tukey tests (*p* < 0.05) were used to analyze the preference of non-viruliferous and viruliferous whiteflies for plants or d-limonene in Y-tube olfactometer experiments. The EPG data exported after software processing were processed using the EPG Excel Data Workbook developed by Sarria et al. [45]. Direct comparisons of feeding behaviors between non-viruliferous and viruliferous whiteflies after d-limonene treatment were made by a non-parametric Mann–Whitney U-test [46]. *p* < 0.05 was regarded as indicating statistically significant differences. Independent samples *t*-test was used to compare the effects of d-limonene treatment on acquisition and transmission of TYLCV by non-viruliferous and viruliferous whiteflies and ANOVAs followed by Tukey tests (*p* < 0.05) used to analyze the relative expression of *BtabOBP*s.

## 3. Results

### 3.1. Key Volatile Compounds in A. graveolens, A. rugosa, and C. sativum and Their Impact on the Preference of B. tabaci

#### 3.1.1. Y-Tube Olfactometer Behavioral Experiments of *B. tabaci* and Plants

**Figure 1:**
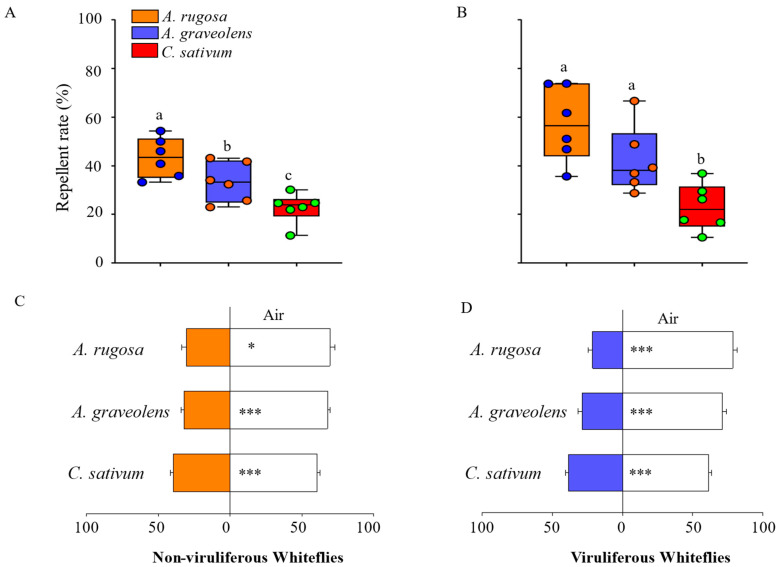
Repellent rate of non-viruliferous *B. tabaci* MED to three plants (**A**) and viruliferous *B. tabaci* MED to three plants (**B**); and preference rate of non-viruliferous *B. tabaci* MED for three plants (**C**) and viruliferous *B. tabaci* MED to three plants (**D**). Each value is the mean ± SEM of six replicates. Different numbers of asterisks (*) and letters above each bar indicate significant differences (*p* < 0.05) among the treatments.

To validate the feasibility of the Y-tube olfactometer behavioral experiments, we assessed the preference of whiteflies for host plants and clean air. The results revealed that viruliferous whiteflies exhibited a preference for healthy tomato over clean air (Figure S3). Subsequently, the repellent effect of *A. graveolens*, *A. rugosa*, and *C. sativum* on healthy and TYLCV-infected whitefly was measured by Y-tube olfactometer behavioral experiments. The results showed that all plants had a strong repellent effect on non-viruliferous and viruliferous whiteflies (Figure 1). In both types of whitefly, the repellent rate of *A. rugosa* was the highest and was 43.42% ± 3.34% in healthy whitefly (Figure 1A) and 57.20% ± 6.27% in TYLCV-infected whitefly (Figure 1B). *A. graveolens* had a relatively weak repellent effect compared with *A. rugosa*, and *C. sativum* was the lowest of the three. Regarding the viruliferous whiteflies, the repellent rates of *A. graveolens* and *C. sativum* were 42.32% and 22.97%, respectively, and were higher than those of non-viruliferous whiteflies (33.40% and 22.70%, respectively). In addition, 30.45% of non-viruliferous whitefly preferred *A. rugosa*, 32.15% preferred *A. graveolens*, and 39.59% preferred *C. sativum* (Figure 1C). The preference trend was consistent with that of the viruliferous whitefly, with the preference rates of 21.40%, 28.84% and 38.52%, respectively (Figure 1D).

#### 3.1.2. Extraction and Identification of Volatiles from Three Plants

The relative content of three plant volatiles was determined: 10 main volatile compounds were found in *A. graveolens* (Table 1), 4 volatile compounds in *A. rugosa* (Table 2), and 10 volatile compounds in *C. sativum* (Table 3). The relative content of d-limonene in *A. graveolens*, *A. rugosa*, and *C. sativum* was 13.7%, 5.7%, and 13.6%, respectively (Table 1, Table 2 and Table 3). In addition, the common volatiles in *A. graveolens* and *C. sativum* were allyl phenoxyacetate, trans-β-ocimene, and (+)-β-selinene. Their relative content in *A. graveolens* was 35.4%, 4.1%, 3.5%, and in *C. sativum*, it was 40.2%, 4.9%, and 3.0% (Table 1 and Table 3). The content of 3-methyl-1-heptene in *A. graveolens* was 0.8%, and myrcene in *C. sativum* was 13% (Table 1 and Table 3).

#### 3.1.3. Y-Tube Olfactometer Behavioral Experiments of *B. tabaci* and D-Limonene

**Figure 2:**
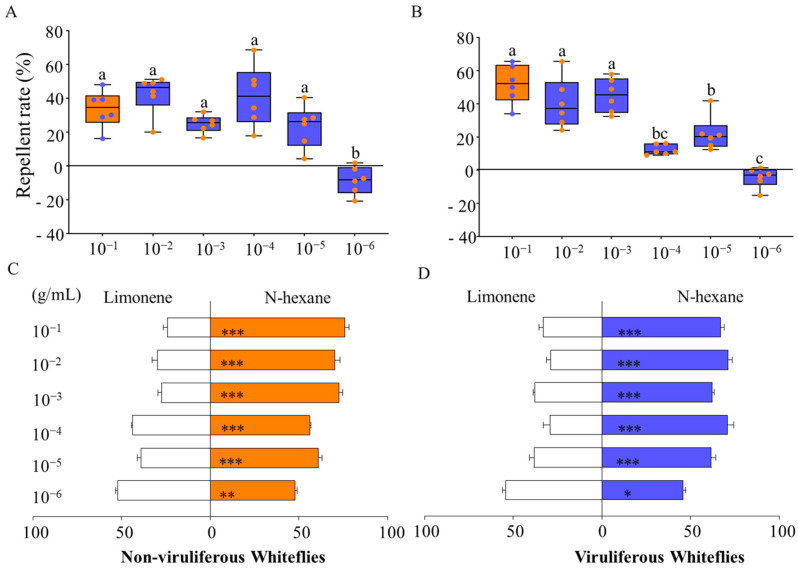
Repellent rate of non-viruliferous *B. tabaci* MED (**A**) and viruliferous *B. tabaci* MED (**B**) to different concentrations of d-limonene and preference rate of non-viruliferous *B. tabaci* MED (**C**) and viruliferous *B. tabaci* MED (**D**) for different concentrations of d-limonene. Different colors indicate different concentrations of d-limonene (specific concentrations are shown on the axis). Each value is the mean ± SEM of six replicates. Different numbers of asterisks (*) and letters above each bar indicate significant differences (*p* < 0.05) among the treatments.

With an increase in the concentration of d-limonene, the preference of non-viruliferous whiteflies exhibited a significant change. When the concentration ranged from 10^−1^ to 10^−5^ g/mL, the repellent rates remained 23.48% to 42.48% (Figure 2A). When the concentration dropped to 10^−6^ g/mL, its repellent effect on *B. tabaci* turned to attraction (Figure 2A,C). The trend of preference in TYLCV-infected *B. tabaci* adults for d-limonene was same as that of uninfected *B. tabaci* adults. The repellent rates remained in the range of 52.00% to 12.27% (Figure 2B). When the concentration decreased to 10^−6^ g/mL, whiteflies preferred d-limonene (Figure 2B,D).

### 3.2. Effects of D-Limonene on Stylet Activities, Acquisition, and Transmission of TYLCV by B. tabaci

#### 3.2.1. Feeding Behavior of *B. tabaci* after D-Limonene Treatment

**Figure 3:**
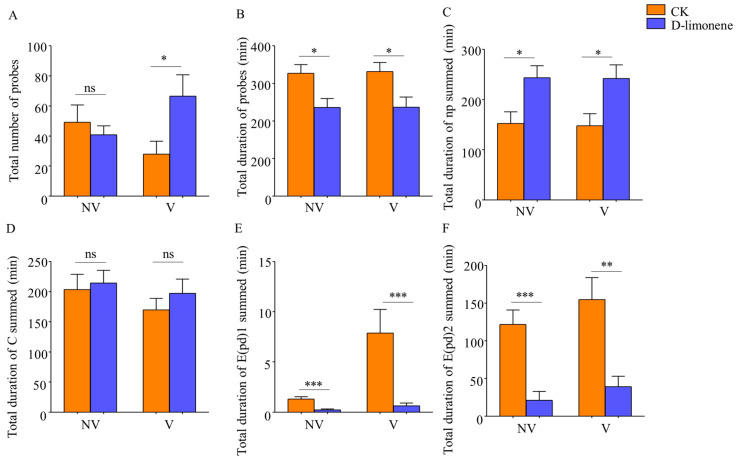
Feeding behavior of non-viruliferous and viruliferous whiteflies after d-limonene treatment. (**A**) Total number of probes. (**B**) Total duration of probes. (**C**) Total duration of np summed. (**D**) Total duration of C summed. (**E**) Total duration of E(pd)1 summed. (**F**) Total duration of E(pd)2 summed. Each value is the mean ± SEM of ten replicates. Different numbers of asterisks (*) above each bar indicate significant differences (*p* < 0.05) among the treatments; no significant difference is denoted as ns.

For non-viruliferous whitefly, the following indicators were significantly smaller compared to the control group: the total duration of probes in the treatment group (F_1,10_ = 7.166, *p* = 0.015) and the total duration of waveforms E1 (F_1,10_ = 19.920, *p* < 0.001) and E2 (F_1,10_ = 19.997, *p* < 0.001) (Figure 3B,E,F). In addition, the duration of waveform np was increased after treatment (Figure 3C). There was no significant difference in the number of probes (F_1,10_ = 0.405, *p* = 0.533) and the duration of waveform C (F_1,10_ = 0.101, *p* = 0.754) (Figure 3A,D).

For viruliferous whitefly, the number of probes (F_1,10_ = 4.928, *p* = 0.04) (Figure 3A) and the duration of waveform np (F_1,10_ = 6.569, *p* = 0.02) were increased in the treatment group compared to the control group (Figure 3C). The total duration of probes (F_1,10_ = 6.569, *p* = 0.02), the time of waveform E1 (F_1,10_ = 9.512, *p* = 0.006), and E2 (F_1,10_ = 12.704, *p* = 0.002) decreased significantly (Figure 3B,E,F). The duration of waveform C (F_1,10_ = 0.787, *p* = 0.387) had no significant difference (Figure 3D). After d-limonene treatment, the duration of waveform np was longer, waveforms E1 and E2 were shortened, and the total probing time was decreased for both infected and healthy whiteflies (Figure 3).

#### 3.2.2. The Acquisition and Transmission of TYLCV after D-Limonene Treatment

**Figure 4:**
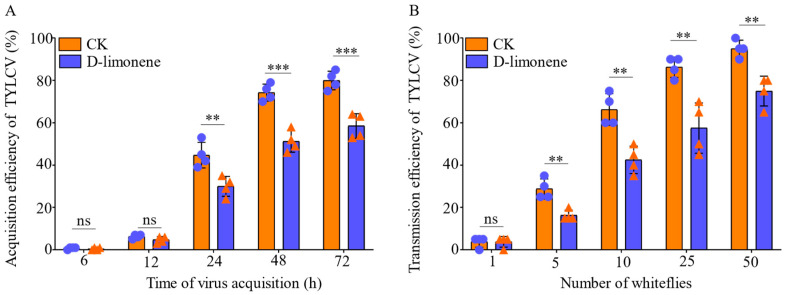
The effects of d-limonene treatment on acquisition (**A**) and transmission (**B**) of TYLCV by non-viruliferous and viruliferous whiteflies. Circles or triangles represent different biological repetitions. Each value is the mean ± SEM of four replicates. Different numbers of asterisks (*) above each bar indicate significant differences (*p* < 0.05) among the treatments; no significant difference is denoted as ns.

Compared with the control treatment, there was no difference in acquisition of TYLCV from 6 to 12 h, and the efficiency of TYLCV acquisition by female adults in the treatment group from 24 to 72 h was significantly reduced to 14.8% (F_1,100_ = 14.9, *p* = 0.008), 23% (F_1,98_ = 49.8, *p* < 0.001), and 21.5% (F_1,95_ = 34.9, *p* = 0.001), respectively (Figure 4A). The transmission rate of TYLCV by 5, 10, 25, and 50 whiteflies showed significant differences compared with the control treatment, which decreased by 13.3% (F_1,20_ = 21.4, *p* = 0.004), 22.7% (F_1,20_ = 23.0, *p* = 0.003), 23% (F_1,20_ = 20.1, *p* = 0.004), and 19.7% (F_1,20_ = 24, *p* = 0.003), respectively (Figure 4B).

### 3.3. Binding Analysis of BtabOBPs Associated with D-Limonene Recognition in B. tabaci

#### 3.3.1. The Relative Gene Expression of BtabOBPs

**Figure 5:**
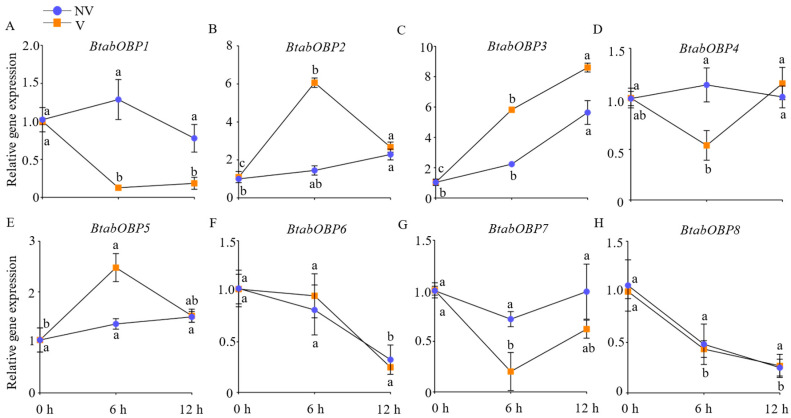
Expression levels of *BtabOBPs* after d-limonene treatment for 0, 6, and 12 h. ((**A**–**H**): *BtabOBP1*-*BtabOBP8*). Values are means ± SEM of three replicates; means with different letters are significantly different at *p* < 0.05.

From 0 h to 12 h, the relative expression of BtabOBP3 in non-viruliferous and viruliferous whiteflies gradually increased with the continuous addition of d-limonene. However, the other seven BtabOBPs did not show a regular trend (Figure 5).

#### 3.3.2. Competitive Fluorescence Binding Assay of BtabOBP3

**Figure 6:**
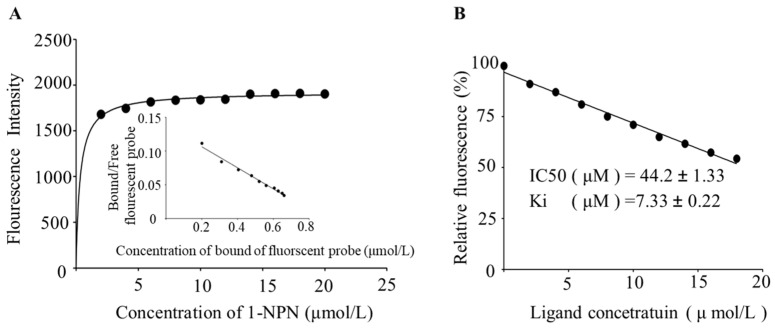
Binding test of BtabOBP3 with d-limonene. (**A**) Linearized spectral data of emission spectrum of 1-NPN and BtabOBP3. (**B**) The relative fluorescence value of d-limonene binding to BtabOBP3. Values are means ± SEM of three replicates.

The Western blot showed that the size of the recombinant protein was 28.57 kDa (Figure S4). The single and bright band of BtabOBP3, which proved the recombinant protein, had an excellent purification effect and can be used for subsequent experiments. As the concentration of 1-NPN increased, the fluorescence intensity of the protein gradually decreased, and the complex gradually increased (Figure S5C). The linearized spectral data showed a good fit (Figure 6A). The dissociation constant of BtabOBP3 and 1-NPN was 5.03 µmol/L. The number of binding sites was 0.994, which indicated that 1-NPN and BtabOBP3 were bound at 1:1. As the concentration of odorant molecules increased, the fluorescence intensity of the protein–probe complex system gradually weakened (Figure S5D). When the relative fluorescence value of the complex decreased to half of the initial value, we calculated IC_50_ = 44.2 ± 1.33 μM, Ki = 7.33 ± 0.22 μM (Figure 6B). The results showed that d-limonene had a strong binding ability to BtabOBP3.

### 3.4. Homology Modeling of BtabOBP3 and Molecular Docking with D-Limonene

**Figure 7:**
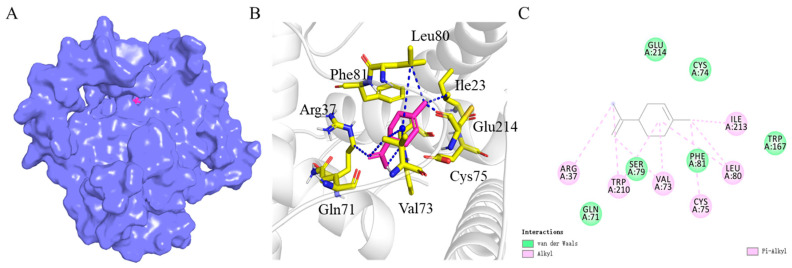
Homology modeling of BtabOBP3 and molecular docking with d-limonene. (**A**) Model diagram of the interaction between d-limonene and BtabOBP3; (**B**) specific binding sites of BtabOBP3 to d-limonene; (**C**) plan of the interaction between d-limonene and BtabOBP3.

The interaction between d-limonene and BtabOBP3 is depicted in the 3D model (Figure 7A) and 2D model (Figure 7C). Hydrophobic interactions exist between Arg37, Leu80, Ile23, Cys75, Val73, and small molecules. The hydrophobic interaction between Arg37 and small molecules was 3.4 A°, and that of Val73 was 3.5 A° and 4.2 A°. The hydrophobic interactions between Leu80, Ile213, Cys75, and small molecules were 3.5 A°, 3.5 A°, and 3.8 A° (Figure 7B).

## 4. Discussion

Virus particles can be transmitted between host plants through vectoring insects. Moreover, TYLCV has been found to be efficiently transmitted through whitefly eggs [7]. With the global invasion of whiteflies, TYLCV threatens the growth of many economically important crops. Therefore, controlling the population of whiteflies on host plants and reducing their ability to transmit TYLCV can safeguard the growth and development of plants. In this study, the repellent effect of three plants on healthy and TYLCV-infected whiteflies was measured by Y-tube olfactometer behavioral experiments, and they had a strong repellent effect (Figure 1). Our results provided a new barrier plant (*A. rugosa*) for the “push–pull” strategy and a possible new target plant for controlling *B. tabaci*. Subsequently, we identified and analyzed three repellent plant volatiles and found that d-limonene was the common volatile of the three plants (Table 1, Table 2 and Table 3). It proved their repellent effect on both healthy and TYLCV-infected whiteflies (Figure 2).

Based on the volatile components of plants, apart from d-limonene, other terpene compounds are present in *A. graveolens*, *A. rugosa*, and *C. sativum*, which may also play a role in the selection of plants by whiteflies. However, we chose d-limonene as the main focus of our study because it exhibited strong repellent effects against whiteflies (Figure 2), and its relative abundance is higher in all three plants (Table 1, Table 2 and Table 3). Tu and Qin (2017) found that β-Myrcene and (E)-Ocimene also had a repellent effect, but the repellent rates were lower than those of d-limonene [47]. Our findings confirmed the original hypothesis that d-limonene plays an important role in the avoidance properties of plants. Furthermore, d-limonene has a high volatility and toxicity to insects but less so to humans, so it is used as a green pesticide [48]. For example, d-limonene acted as an insect repellant to control the population of *B. tabaci* and reduced their feeding on host plants [49]. Also, the main components of *Zanthoxylum bungeanum* essential oil were d-limonene and linalool, which had a good toxicity effect on insects [50]. In summary, d-limonene can play an effective role in controlling whiteflies and is a functional volatile. Furthermore, spraying d-limonene on plants can be considered in future applications, and the preference of whiteflies for tomatoes treated with d-limonene (treatment) or n-hexane (control) was determined to investigate whether the repellent effect persists under natural crop conditions. Our results indicated that spraying d-limonene on the surface of tomatoes still has a repellent effect (Figure S6). It is worth noting that the repellent effect of d-limonene was concentration-dependent, and appropriate spraying concentrations should be considered in future field applications, considering factors such as planting area and host plant growth, to achieve optimal control efficacy.

Volatiles affect the physiological processes of insects and are also the main basis for selecting target plants for barrier crop protection. Previous studies have found that cucumber intercropping with celery (*A. graveolens*) could reduce the number and oviposition of *B. tabaci* on cucumbers [47]. Results from our study showed that spraying of d-limonene on tomato leaves affected the feeding behavior of *B. tabaci*. After d-limonene treatment, the duration of waveform np (non-feeding) was longer, waveform E1 and E2 was shortened, and the total probing time was decreased for both infected and healthy females (Figure 3). According to the characteristics and mechanism of TYLCV transmission by whiteflies, they ingest virions while feeding on phloem sap, leading to f phytotoxic disorders [51]. Regarding whiteflies, TYLCV particles travel up the salivary glands into the gut and are further transported to the hemolymph, then to the salivary glands, and finally back into the new plants during the next feeding [52]. Therefore, phloem feeding is the key for whiteflies to acquire and spread TYLCV. Reducing the occurrence of this behavior can significantly reduce the acquisition and transmission efficiency of the virus. In addition, female whiteflies were more capable of transmitting the virus than males [53]. Our results on feeding behavior combined with the acquisition and transmission of TYLCV confirmed that the decreased duration of waveforms E1 and E2 significantly reduces the transmission efficiency of TYLCV. The results suggested that d-limonene can affect the acquisition and transmission of females by changing feeding, which meant that d-limonene might play an anti-feeding role in insect control. It is noteworthy that Jiang et al. (2000) reported a strong correlation between the duration of waveform E1 and E2 and TYLCV inoculation, especially with E1 playing a pivotal role. Furthermore, the total number of pds was also associated with virus inoculation. However, executing E2 waveforms was not crucial for virus acquisition, as the results indicated a negative correlation with virus inoculation. This phenomenon seemed difficult to explain and suggested that the whiteflies might not acquire sufficient virus particles despite multiple probing events. Therefore, we focused on analyzing only six parameters related to non-probing waveforms and phloem-feeding waveforms for feeding analysis [54].

Next, the molecular mechanism of recognition of d-limonene by whitefly was explored. OBPs are important components of the olfactory system and play a key role in the recognition of signals in *B. tabaci*, and their relationship with volatiles was also revealed [25,29]. For example, BtabOBP1 played a key role in the recognition of *R*-curcumene, causing differences in *B. tabaci* preference for wild and cultivated tomatoes [55]. BtabOBP1 and BtabOBP4 could bind β-ionone and affect the localization of *B. tabaci* to egg-laying sites [56]. Our study showed that the relative expression of BtabOBP3 in non-viruliferous and viruliferous whiteflies gradually increased with the continuous addition of d-limonene by qPCR (Figure 5). Combined with the results of fluorescence competition (Figure 6, Figures S4 and S5), this indicates that BtabOBP3 played a role in the olfactory response of *B. tabaci* MED to d-limonene (Figure 5). In addition, the binding sites of BtabOBP3 and d-limonene were predicted by molecular docking (Figure 7). BtabOBP3 has been found to be highly expressed in the head and combined with odorant molecules, such as β-ionone, trans-cinnamaldehyde, trans-2-hexenal, linalool, naphthalene, cedrol, 1,8-cineole, and β-citronellol [56]. Our results confirmed that the recognition of d-limonene by *B. tabaci* was also related to *BtabOBP3*. *BtabOBP3* played a key role in the odor recognition process of *B. tabaci*. In addition, the transmission rate of ToCV was reduced by 83.3% after feeding *dsBtabOBP3*, so *BtabOBP3* was likely to become an essential target for controlling *B. tabaci* [57]. D-limonene has high volatility and toxicity to insects but less so to *Homo sapiens* (humans), so it is used as a green pesticide [48]. For example, d-limonene acted as an insect repellant to control the population of *B. tabaci* and reduced their number on host plants [49]. The main components of *Zanthoxylum bungeanum* essential oil were d-limonene and linalool, which had an excellent toxicity effect on *Tribolium castaneu* [50].

## 5. Conclusions

Our results confirmed that d-limonene is an important functional volatile, showing a potential contribution against viral infections with potential implications for developing effective TYLCV control strategies.

## Figures and Tables

**Table 1:** Components and relative contents of volatiles in *Apium graveolens*.

Number	Compound	CAS	Formula	Relative Content (%)
1	Allyl phenoxyacetate	7493-74-5	C_11_H_12_O_3_	35.4
2	o-Phenylenediamine	95-54-5	C_6_H_8_N_2_	26.6
3	D-Limonene	5989-27-5	C_10_H_16_	13.7
4	Trans-ligustilide	100036-59-8	C_12_H_14_O_2_	5.7
5	1-Methoxy-3-(2-hydroxyethyl) nonane	70928-44-8	C_12_H_26_O_2_	5.2
6	Trans-β-Ocimene	3779-61-1	C_10_H_16_	4.1
7	(+)-β-Selinene	17066-67-0	C_15_H_24_	3.5
8	4-Ethylbenzoic acid, allyl ester	100029-33-7	C_12_H_14_O_2_	3.1
9	1-Acetyl-2-phenylhydrazine	114-83-0	C_8_H_10_N_2_O	1.9
10	3-Methyl-1-heptene	4810-09-7	C_8_H_16_	0.8

**Table 2:** Components and relative contents of volatiles in *Agastache rugosa*.

Number	Compound	CAS	Formula	Relative Content (%)
1	Pulegone	15932-80-6	C_10_H_16_O	85.3
2	D-Limonene	5989-27-5	C_10_H_16_	5.7
3	Isopulegone	29606-79-9	C_10_H_16_O	4.5
4	1,3,4-Trimethylcyclohex-3-enecarbaldehyde	40702-26-9	C_10_H_16_O	4.5

**Table 3:** Components and relative contents of volatiles in *Coriandrum sativum*.

Number	Compound	CAS	Formula	Relative Content (%)
1	Allyl phenoxyacetate	7493-74-5	C_11_H_12_O_3_	40.2
2	D-Limonene	5989-27-5	C_10_H_16_	13.6
3	Myrcene	123-35-3	C_10_H_16_	13.0
4	1,2-Benzenediamine	95-54-5	C_6_H_8_N_2_	12.9
5	Trans-β-Ocimene	3779-61-1	C_10_H_16_	4.9
6	3-Methylcyclohex-4-ene-1,2-dicarboxylic acid	40469-16-7	C_9_H_12_O_4_	4.8
7	(+)-β-Selinene	17066-67-0	C_15_H_24_	3.0
8	2,3-Dimethylnonadecane	75163-99-4	C_21_H_44_	1.5
9	3-Methylphenol acetate	122-46-3	C_9_H_10_O_2_	3.0
10	1-Acetyl-2-phenylhydrazine	114-83-0	C_8_H_10_N_2_O	3.0

## Data Availability

Data are contained within the article and Supplementary Materials.

## Supplementary Materials

The following supporting information can be downloaded at: https://www.mdpi.com/article/10.3390/v16020300/s1, Table S1: Primers used in this study; Figure S1: Tomato plants. (A) Healthy tomato leaves. (B) TYLCV-infected tomato leaves; Figure S2: Female and male *B. tabaci* under an in stereo microscope; Figure S3 Preference of viruliferous *B. tabaci* MED for air vs. tomato; Figure S4: Expression tests of the target protein; Figure S5: Spectra of BtabOBP3 binding test with d-limonene. Figure S6: Preference of viruliferous *B. tabaci* MED for tomatoes mixed with 10^−2^ g/mL d-limonene vs. tomatoes mixed with n-hexane.
