# Supplementary material for: D-Limonene Affects the Feeding Behavior and the Acquisition and Transmission of Tomato Yellow Leaf Curl Virus by Bemisia tabaci
Source: Viruses. 2024 Feb 15;16(2):300. doi: 10.3390/v16020300 (PMC10891612; doi:10.3390/v16020300)
Supplement: Supplementary file 1 [file viruses-16-00300-s001.zip › viruses-2848459-Supplementary.pdf]

Table S1. Primers used in this study

Gene name	Purpose	Primer Sequence (5 ′– 3′)
TYLCV-F	Detection of TYLCV	ATGTCGAAGCGACCAGGC
TYLCV-R		GATGCGTATTTTCATAGTTGCATATACTGG
Actin-F	*B. tabaci* reference genes	CGGTGATTTCCTTCTGCATT
Actin-R		ACCGCAAGATTCCATACCC
EF-1α-F	*B. tabaci* reference genes	TAGCCTTGTGCCAATTTCCG
EF-1α-R		CCTTCAGCATTACCGTCC
BtabOBP1-F	*BtabOBP1* RT-qPCR	AAGTGCTTGACGGATTATTAC
BtabOBP1-R		GCATCATATTATCGCAGTGT
BtabOBP2-F	*BtabOBP2* RT-qPCR	CAACAACCCCTCAACCGACA
BtabOBP2-R		TCATGTCAGGTGTCAGAAGGC
BtabOBP3-F	*BtabOBP3* RT-qPCR	CTATCTCGGTTCAGTTCCA
BtabOBP3-R		TGTCTTTCCACTCGCTAT
BtabOBP4-F	*BtabOBP4* RT-qPCR	GTTTCTTGGAGTGCGTTTA
BtabOBP4-R		TCATCATCATCAGCCTCTT
BtabOBP5-F	*BtabOBP5* RT-qPCR	AAGTAAAGGCTGTGGATGA
BtabOBP5-R		CGAGTAATAGTTGTTGTCTTGA
BtabOBP6-F	*BtabOBP6* RT-qPCR	GTAGCAATACAGGTGGAGA
BtabOBP6-R		ATGACACTCTTGACATTAGC
BtabOBP7-F	*BtabOBP7* RT-qPCR	TCGAATCAGATGCAGAGGGTG
BtabOBP7-R		TATCCGGGGGACTCATTCCA

BtabOBP8-F	*BtabOBP8* RT-qPCR	TGATGGCGTGTCTTATGA
BtabOBP8-R		CTGAGGTTGAGTGCTGTA

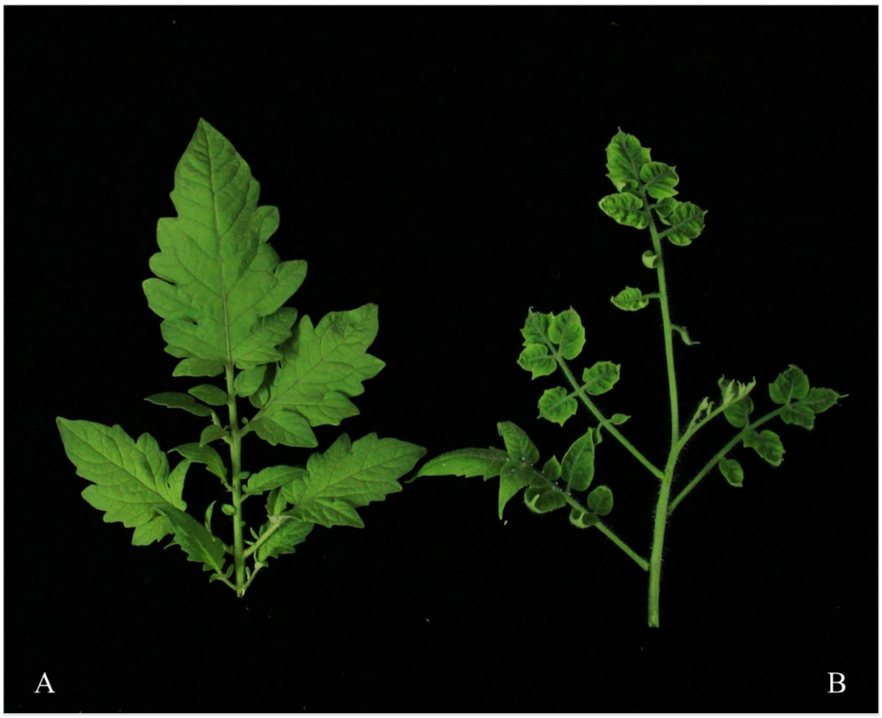

Figure S1 Tomato plants. (A) Healthy tomato leaves. (B) TYLCV-infected tomato leaves.

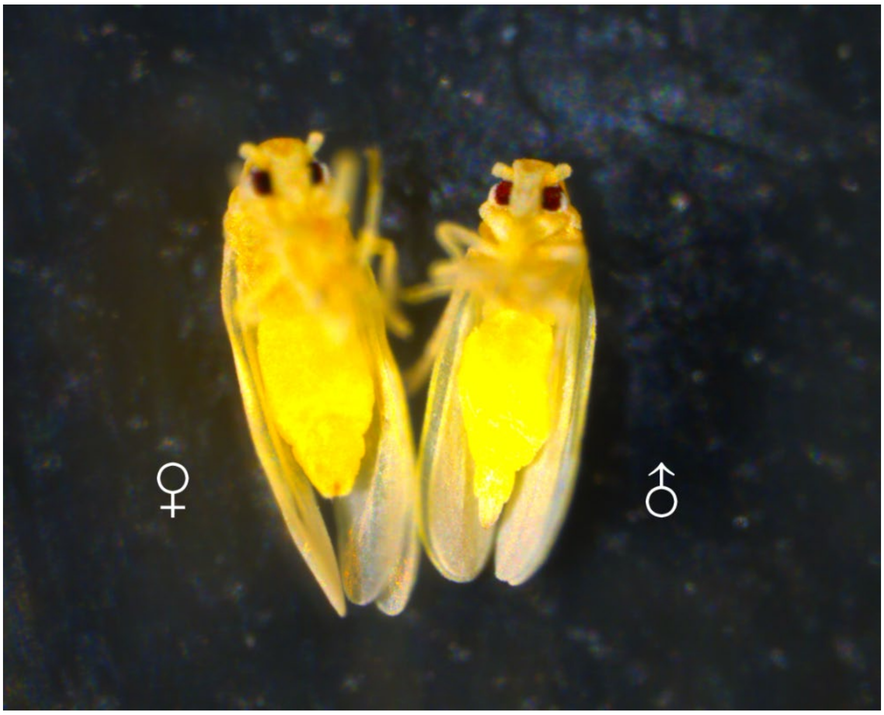

Figure S2 Female (♀) and male (♂) *B. tabaci* under an in stereo microscope.

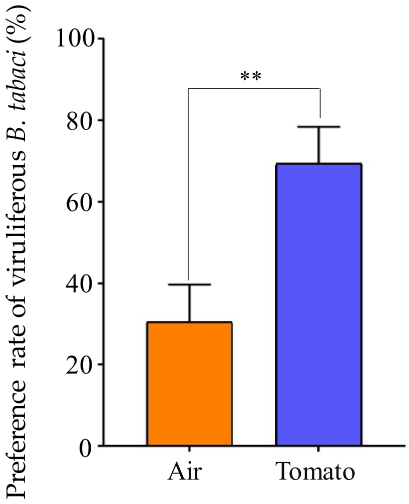

Figure S3 Preference of viruliferous *B. tabaci* MED for air vs. tomato. Values are means ± SEM. Different number of asterisk (*) and letters above each bar indicate significant differences (P<0.05) among the treatments.

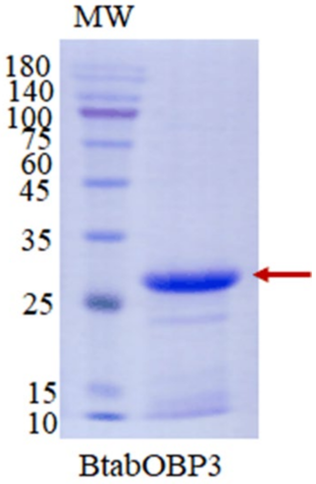

Figure S4 Expression tests of the target protein.

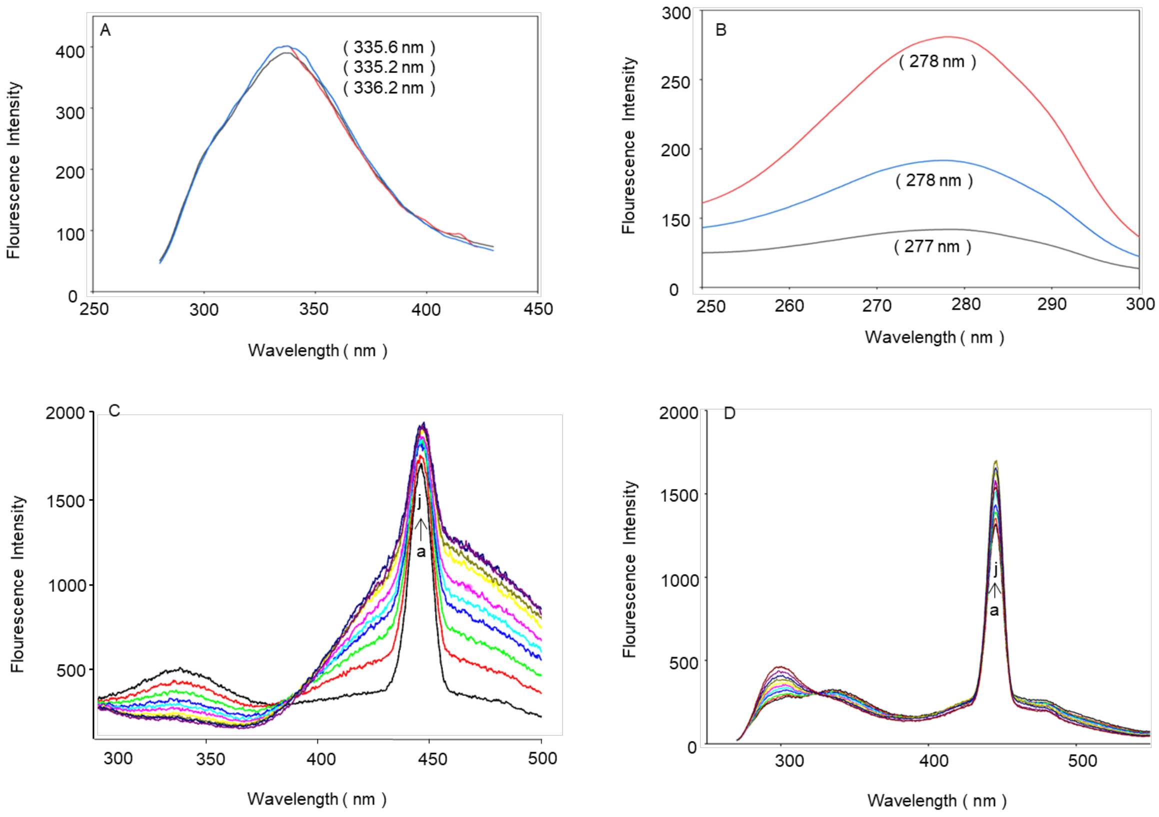

Figure S5 Spectra of BtabOBP3 binding test with d-limonene. (A) Determination of the optimum emission wavelength of BtabOBP3. (B) Determination of the optimum excitation wavelength of BtabOBP3. (C) Emission spectrum of 1-NPN and BtabOBP3. (D) D-limonene competes for 1-NPN emission spectra.

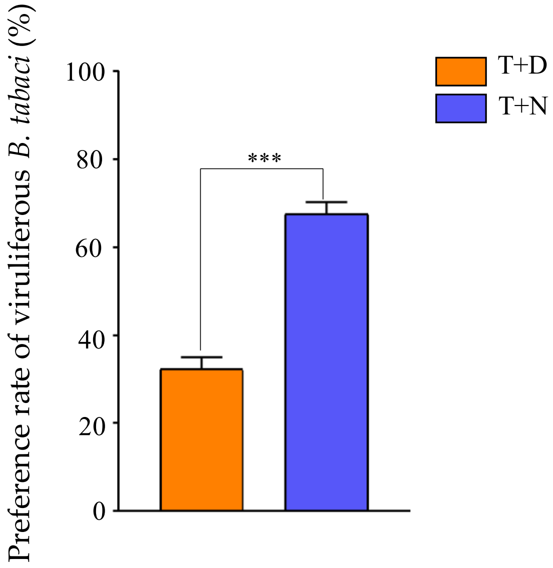

Figure S6 Preference of viruliferous *B. tabaci* MED for tomatoes mixed with 10^{-2} g/mL d-limonene (T+D) vs. tomatoes mixed with n-hexane (T+N). Values are means ± SEM. Different number of asterisk (*) and letters above each bar indicate significant differences (P<0.05) among the treatments.
